# Genetic Basis of Self-Incompatibility in the Lichen-Forming Fungus *Lobaria pulmonaria* and Skewed Frequency Distribution of Mating-Type Idiomorphs: Implications for Conservation

**DOI:** 10.1371/journal.pone.0051402

**Published:** 2012-12-07

**Authors:** Garima Singh, Francesco Dal Grande, Carolina Cornejo, Imke Schmitt, Christoph Scheidegger

**Affiliations:** 1 Biodiversity and Conservation Biology, WSL Swiss Federal Research Institute, Birmensdorf, Switzerland; 2 Biodiversity and Climate Research Centre (BiK-F), Senckenberg Gesellschaft für Naturforschung, Frankfurt am Main, Germany; 3 Institute of Ecology, Evolution and Diversity, Goethe Universität, Frankfurt, Germany; Soonchunhyang University, Republic of Korea

## Abstract

Fungal populations that reproduce sexually are likely to be genetically more diverse and have a higher adaptive potential than asexually reproducing populations. Mating systems of fungal species can be self-incompatible, requiring the presence of isolates of different mating-type genes for sexual reproduction to occur, or self-compatible, requiring only one. Understanding the distribution of mating-type genes in populations can help to assess the potential of self-incompatible species to reproduce sexually. In the locally threatened epiphytic lichen-forming fungus *Lobaria pulmonaria* (L.) Hoffm., low frequency of sexual reproduction is likely to limit the potential of populations to adapt to changing environmental conditions. Our study provides direct evidence of self-incompatibility (heterothallism) in *L. pulmonaria*. It can thus be hypothesized that sexual reproduction in small populations might be limited by an unbalanced distribution of mating-type genes. We therefore assessed neutral genetic diversity (using microsatellites) and mating-type ratio in 27 lichen populations (933 individuals). We found significant differences in the frequency of the two mating types in 13 populations, indicating a lower likelihood of sexual reproduction in these populations. This suggests that conservation translocation activities aiming at maximizing genetic heterogeneity in threatened and declining populations should take into account not only presence of fruiting bodies in transplanted individuals, but also the identity and balanced representation of mating-type genes.

## Introduction

Sexual reproduction is an important factor for introducing genetic variation in populations, thus providing the basis for long-term adaptation and population survival [1]. Reshuffling gene combinations in sexual reproduction can potentially improve the adaptability of populations by producing progeny with enhanced abilities to cope with changing environmental conditions. In a varying habitat, sexual populations thus have higher chances of survival and evolutionary potential than asexual populations [2]. Asexual reproduction, in contrast, leads to genetic uniformity and is favorable for well-adapted populations in stable habitats as it preserves locally adapted genotypes. Genetic uniformity is the basis of vulnerability to epidemics and to biotic and abiotic stress [3] as it limits the ability of species to respond to these threats in both short and long terms. A population with low genetic diversity may have a low adaptive potential.

Lichens, symbioses between a fungus and one or more algal photobiont species, have often developed a complex reproductive strategy that combines clonal and sexual reproduction [4]–[7]. The costs and benefits of the two reproductive modes and hence the frequency of their occurrence is governed by specific environmental conditions and the ecological strategy of the species [8]–[10]. Because of their sensitivity to environmental and habitat changes, a large number of lichen species have declined in anthropogenically influenced habitats [11]–[13]. Along land-use gradients in an European study, reproductive modes explained a significant part of the lichen community with species with sexual reproduction being more frequent in open, intensively managed agricultural land and species with relatively large, vegetative propagules were restricted to forest landscapes with a lower level of disturbance frequency [14]. By alternating clonal and sexual mode of reproduction the best of the traits are preserved and reshuffled to generate more suitable gene combinations resulting in locally adapted genotypes [2].

In the fungi, almost the entire sexual cycle starting from the recognition of a potential mate to the development of sexual structures, is regulated by a specialized region of the genome known as the mating-type (*MAT*) locus [15]–[17]. In ascomycetes, which represent the majority of fungal symbionts in lichens, sexual reproduction is controlled by two alternative forms, *MAT1-1* and *MAT1-2*, of the single regulatory *MAT* locus [5], [16]. The alternative forms of the *MAT* locus are referred to as “idiomorphs” rather than alleles [18]–[19]. In spite of being involved in the common process of “regulating sexual reproduction”, the sequence and number of encoded proteins and transcription factors vary strikingly between the two mating systems, bearing no obvious allelic relationship to one another. One of the characteristics of *MAT* genes is the presence of conserved DNA binding motifs in the encoded proteins, which might serve as transcription regulators [16], [19]. For example, *MAT1-1* encodes a protein with conserved alpha box motif, and MAT1-2 protein contains conserved DNA binding motif called HMG box [18]–[20]. However it has been shown that the alpha-box and the HMG domains are evolutionary related and share similar structural features in-spite of low sequence conservation [21]. These regions display similar sequence features in different species that can be targeted by degenerate PCR approaches [22]–[25].

The ascomycetes mating system may be further classified as homothallic or heterothallic [18]. In homothallic species each strain is self-fertile, requiring only one individual and a mating type cannot be defined. In heterothallic species each haploid individual harbors either *MAT1-1* or *MAT1-2*, thus being self-incompatible. Sexual reproduction in heterothallic species is accomplished only when gamete nuclei come from parents with different mating types. The relative proportion of mating types can therefore have a critical influence on the reproductive potential of the population [26]. Recombination in a population of a heterothallic species, having a significantly unbalanced MAT isolates ratio, may be restricted because of the paucity of compatible sexual partners/spores. This effect may be aggravated by limited dispersal capacity of the species and patchy distribution of suitable habitats. In some cases, fixation of a single mating type in certain areas may impose an obligate asexual mode of reproduction in functionally heterothallic taxa (e.g., *Magnaporthe grisea* [27]–[28], *Aspergillus flavus* [29]).

The assessment of genetic variation is widely used in preserving threatened organisms particularly in species with small and isolated populations. Despite the critical influence of sexual reproduction on population genetic structure, long-distance dispersal and evolutionary-adaptive potential of a species, mating systems of lichen-forming fungi remain relatively unexplored. However, a broad understanding of mating systems and their distribution in natural populations is therefore essential for devising sound conservation measures.

In this study we investigate mating-type loci in the epiphytic lichen-forming fungus *Lobaria pulmonaria (L.) Hoffm.* This is one of the best-studied lichen species [30], concerning its ecology [31]–[32], population genetics [33]–[34] and photobiont selection [35]. It has a wide geographic distribution covering major parts of Europe, Asia, North America and Africa. However it has suffered a substantial decline in European lowlands due to habitat destruction and environmental pollution, and the species is considered threatened throughout Europe and in many parts of North America [36]–[37]. Previous studies [38] postulated *L. pulmonaria* to be heterothallic. However heterothallism in *L. pulmonaria* has not yet been demonstrated by molecular analyses.

Microsatellites are the marker of choice for estimating genetic diversity and population structure of highly clonal organisms such as lichens. The high mutation rate of microsatellite loci and thus high variability gives them a far greater resolving power than previous, sequence- based method. They are valuable tools for detecting linkage disequilibrium associated with the absence of sexual reproduction [35]. Recently, fungal and algal species-specific highly variable microsatellite markers were used to infer the genetic structure of the *L. pulmonaria* symbiosis and to infer the relative contribution of sexual reproduction to the genetic composition of lichen thalli in natural populations [35], [39]–[40].

Our objectives here were to 1. Determine the mating system (homothallic/heterothallic) in the lichen model organism *Lobaria pulmonaria*, 2. Assess the distribution and frequencies of the two mating types in 27 populations of *L. pulmonaria* with newly developed, species-specific mating-type multiplex markers. 3. Investigating the genetic structure and linkage disequilibrium pattern using fungal and algal-specific microsatellite markers for these populations.

## Materials and Methods

### Cultivation and DNA Isolation

Mycelia of two *L. pulmonaria* specimens (vouchers of the Herbarium of the Swiss Federal Research Institute WSL, Birmensdorf: 17061, Switzerland, in this study F1; 10161, Scotland, in this study F2) were cultivated according to Denison (2003) [41], with some modifications. A thallus with apothecia was dried over silica gel and apothecia were collected on filter paper moistened with sterilized water and were then placed on the caps of inverted Petri dishes containing sterile medium of 1% alpha-cyclodextrin (Sigma-Aldrich) and 1.5% corn meal agar (BD). Spores were discharged upward to the medium within 1–2 days and started to germinate after three days. Caps with filter papers and apothecia were then replaced by new sterile ones. After two weeks of growth, axenic mycelia were transferred to new Petri dishes. Germination and growth took place at 16°C in complete darkness for three months. For each fungal culture, ca. 17 mg of the mycelia grown from hundreds of germinated spores, were harvested under sterile conditions and pooled in an ice-cooled eppendorf tube containing 30 ml of lysis buffer AP1 (Qiagen). The mycelia in the tube were frozen before disruption of cell material with a mixer mill (MM300, Retsch). DNA was isolated using DNeasy Plant Mini Kit (Qiagen, Venlo, The Netherlands) according to the manufacturer’s instructions (Mini Protocol).

Photobiont DNA from *L. pulmonaria* was extracted as described in [42]. The extraction was done with GenElute Plant Genomic DNA Miniprep Kit (Sigma-Aldrich) according to the manufacturer’s instructions.

In addition, total genomic DNA, containing both fungal and algal DNA, was extracted using DNeasy Plant Mini Kit (Qiagen) according to the manufacturer’s recommendations. This involved isolation from single lobes of *L. pulmonaria* specimens from populations previously described in [35], [40] (see Table 1).

**Table 1:** Information on the populations used in this study. See footnotes below for definition of terms.

Country	Pop	N	FG	FG:N	MAT1-1: MAT1-2	X2	P-value	I^A^	A	H	AG:N	LG:N	% CF	% CL
Bulgaria	BuD^1^	28	14	0.50	14∶14	0.034	0.8527	0.0773	2.80434	0.36463+/−0.34084	0.64	0.68	50	32
France	FV^1^	35	17	0.49	21∶14	1.4	0.2367	0.1096	2.84749	0.39463+/−0.33374	0.60	0.60	51.42	40
Germany	**DS1** 1	71	21	0.30	27∶44	4.07	0.0436	0.2635	3.49557	0.56377+/−0.28593	0.27	0.32	70.42	68
Greece	GR1^1^	34	25	0.74	15∶19	0.471	0.4927	0.0119	3.45839	0.46701+/−0.33606	0.85	0.85	26.47	18
	GR3^1^	42	27	0.64	19∶23	0.381	0.5371	0.0222	3.18962	0.43132+/−0.36167	0.83	0.83	35.71	17
	**GRR** 1	35	8	0.23	30∶5	17.857	<0.0001	0.3987	2.53574	0.36356+/−0.27956	0.23	0.34	77.14	66
Ireland	IRK^1^	35	27	0.77	13∶22	2.314	0.1282	0.0344	3.4361	0.43741+/−0.39703	0.86	0.91	22.85	9
Norway	**NA** 1	17	9	0.53	3∶14	7.118	0.0076	0.354	3.29721	0.49658+/−0.26437	0.47	0.53	47.05	47
	NT^1^	31	18	0.58	13∶18	0.806	0.3692	0.1607	3.63831	0.51839+/−0.21392	0.55	0.68	41.93	32
	**MO1** 1	19	13	0.68	3∶16	8.895	0.0029	0.1121	3.82905	0.584+/−0.19880	0.79	0.84	31.57	16
Portugal	PO^1^	38	13	0.34	23∶15	1.684	0.1944	0.4204	3.12632	0.51596+/−0.26134	0.47	0.50	65.57	50
Russia	**RTL** 3	55	47	0.85	35∶20	4.091	0.0431	0.0372	3.02682	0.45137+/−0.33036	0.58	0.93	14.54	9
	RVZ^3^	16	14	0.88	6∶10	1	0.3173	0.0148	3.93858	0.45781+/−0.33227	0.69	0.88	12.50	13
	RXS^3^	69	62	0.90	33∶36	0.13	0.718	0.0099	3.88675	0.43174+/−0.32194	0.71	0.93	10.14	7
South Africa	SA5^2^	21	17	0.81	9∶12	0.429	0.5127	0.029	3.00305	0.39123+/−0.30930	0.95	0.95	19.04	5
	**SA6** 2	21	17	0.81	4∶17	8.048	0.0046	0.0915	3.30308	0.40247+/−0.34825	0.90	0.90	19.04	10
	SA10^2^	17	13	0.76	12∶5	2.882	0.0896	0.0934	2.94845	0.41834+/−0.32573	0.82	0.82	23.52	18
Spain	**EG** 1	21	18	0.86	2∶19	13.762	0.0002	0.0853	4.07909	0.53849+/−0.31368	0.86	0.86	14.28	14
	ES^1^	63	20	0.32	32∶31	0.016	0.8997	0.18	2.85694	0.40587+/−0.23356	0.37	0.41	68.25	59
	**EGR** 1	9	5	0.56	1∶8	5.444	0.0196	0.1648	2.625	0.3621+/−0.27290	0.67	0.67	44.44	33
Switzerland	**B** 1	43	12	0.28	14∶29	5.233	0.0222	0.1415	2.8498	0.41042+/−0.30149	0.42	0.44	72.09	56
	CHM^1^	39	19	0.49	18∶21	0.231	0.631	0.1135	3.10305	0.43997+/−0.30474	0.44	0.49	51.28	54
	**CHN** 1	31	14	0.45	7∶24	9.323	0.0023	0.0766	2.8569	0.34557+/−0.32137	0.45	0.48	54.83	52
	CHB^1^	42	12	0.29	22∶20	0.095	0.7576	0.0781	2.6866	0.34747+/−0.35883	0.31	0.33	71.42	67
	**CHV1** 1	23	11	0.48	17∶6	5.261	0.0218	0.1663	2.48463	0.28048+/−0.34309	0.52	0.57	52.17	43
	**CHS** 1	36	4	0.11	31∶5	18.778	0.0001	0.4663	1.59751	0.13394+/−0.12625	0.25	0.25	88.88	75
Ukraine	**CA** 1	42	36	0.86	14∶28	4.667	0.0308	0.0729	3.77263	0.47446+/−0.32374	0.88	0.90	14.28	10

1 ([40] - Appendix S1b);

2 ([35] - Table S1]);

3 [47].

### 454 Pyrosequencing of *L. pulmonaria*

The DNA of the mycobiont culture of *L. pulmonaria* was selected to be shotgun sequenced (1/4^th^ run) using a Roche 454 Genome Sequencer FLX with the Titanium Sequencing kit XLR 70 at Microsynth AG (Balgach, Switzerland). The 454 sequencing was done with the culture 10161 (Great Britain, Scotland, W 05°01' 23.6" N 36°41'47.7"). We obtained 73.2 Mbp of raw sequence data, from the 1/4 run of 454 sequencing, containing 181’541reads/sequences with an average length of 363 bp. The number of assembled sequences was 139937 with an average length of 369 bp. The raw sequences represented large numbers of individual sequence reads, which were then assembled into contigs. A total of 181541 reads were assembled into 41692 contigs with an average length of 685 bp.

### Degenerate Primer PCR and Inverse PCR

The conserved regions of *MAT1-1* and *MAT1-2* genes of the fungal DNA were first amplified using several sets of degenerate primers (Table 2). Based on these sequences, species-specific primer pairs were designed for sequencing out and population analysis. The flanking regions of *MAT1-2* were amplified by inverse PCR [43]. To prepare for inverse PCR, genomic DNA was digested with four restriction enzymes having recognition sites outside the known region MspI, HhaI (Invitrogen, Switzerland) at 37°C for three hours (in 10 µl volume containing 1X buffer, 5U of enzyme and 50 ng of DNA), followed by 20 mins heat inactivation of the enzyme at 60°C or 65°C. The digested DNA was then allowed to self-ligate overnight at 16°C in 300 µl reaction volume with 1X ligation buffer and 20 U T4 DNA ligase, followed by ethanol precipitation. Precipitated DNA was then re-digested overnight at 37°C with a six-cutter enzyme- XbaI (Invitrogen Switzerland) having recognition site within the known region. Reaction was performed in 10 µl having 1X buffer and 3 U enzyme. The product obtained was then used as a template for inverse PCR.

**Table 2:** List of primers used in the study.

Primer	Sequence	Expected product size	Target	Reference
ChHMG1	CCYCGYCCTCCTAAYGCNTAYAT			
ChHMG2	CGNGGRTTRTARCGRTARTNRGG	300 bp	HMG box (*MAT1-2*)	[22]
NcHMG1	CCYCGYCCYCCYAAYGCNTAYAT			
NcHMG2	CGNGGRTTRTARCGRTARTNRGG	300 bp	HMG box (*MAT1-2*)	[22]
RsMAT-1F	ACRTCCTTCTCTGTCATGCTCG			
RsMAT-1R	TAYYTGATGAACTAYCCTATAC	560 bp	Alpha box (*MAT1-1*)	[55]
MAT1-2_invFP1	ACCCCGACCTCCATAACAAT			
MAT1-2_invRP1	GGGTGATCCTCCATATGCTT	179 bp	*L. pulmonaria* HMG box (*MAT1-2)*	This study
MAT1-2invFP2	CTCACCCCGACCTCCATAAC			
MAT1-2invRP2	TGCTTCTTCTTGAGGTCATCC	181 bp	*L. pulmonaria* HMG box *(MAT1-2*)	This study
Lpul_MAT1_F	ATGGCATTCCGTGGTAAGTC			
Lpul_MAT1_R	TCGACAGTACGACGCAACAT	390 bp	*L. pulmonaria* Alpha box (*MAT1-1)*	This study
Lpul_MAT1_F2	GGAAGCAATGGTTCTAGTGTCA			
Lpul_MAT1_R2	TAGCAAGGAACGCATCAAGA			This study
TAIL_Alpha_AD1	AGWGNAGWANCAWAGG		Alpha box (*MAT1-1*)	[45]
TAIL_Alpha_AD2	TCSTNCGNACNTWGGA		Alpha box (*MAT1-1*)	[45]
TAIL_HMG_AD1	NGTCGASWGANAWGAA		HMG box (*MAT1-2*)	[45]
TAIL_HMG_AD5	TGYCCNAAYMGNYT		HMG box (*MAT1-2)*	[45]
TAIL_HMG_AD5	TTRTTNCCRTANCCDAT		HMG box (*MAT1-2*)	[45]
*MAT1-1*-T-UP1	GATGAAGAAAGAGAATGGG		Alpha box (*MAT1-1*)	This study
*MAT1-1*-T-UP2	GATACTTGTACTGTTGCGCTG		Alpha box (*MAT1-1*)	This study
*MAT1-1*-T-UP3	GTGAACGCTGTGTTGAAGTG		Alpha box (*MAT1-1*)	This study
MAT1-1-T-DN1	TGGAAGATAGCGCCGCCGA		Alpha box (*MAT1-1*)	This study
MAT1-1-T-DN2	CTAACGGCCGAAGCTGTGAT		Alpha box (*MAT1-1*)	This study
MAT1-1-T-DN3	ATGACACAGTGAACCCTGGA		Alpha box (*MAT1-1*)	This study
MAT1-2-T-UP1	TCGGGGTGAGCTCTCACTAT		Alpha box (*MAT1-1*)	This study
MAT1-2-T-UP2	CCTCGTTCTTCCACTGCTTT		HMG box (*MAT1-2*)	This study
MAT1-2-T-UP3	GGGTGATGGTGTTGACGATA		HMG box (*MAT1-2*)	This study
MAT1-2-T-DN1	AAGAAGAGGCTGTGATCTCT		HMG box (*MAT1-2*)	This study
MAT1-2-T-DN2	CACCCCATAGTCAAAGAAGCT		HMG box (*MAT1-2*)	This study
MAT1-2-T-DN3	CACCCCATAGTCAAAGAAGCT		HMG box (*MAT1-2*)	This study

All amplifications were performed with 20 ng of DNA extract in a total volume of 50 µl containing 18 µl of JumpStart REDTaq ReadyMix PCR Reaction Mix (Sigma-Aldrich), 500nM of each degenerate primer or 250 nM of the specific ones. The following cycling conditions were used for *MAT1-1* with degenerate primers: (1) 2 min 94°C; (2) 40 cycles of 30 sec 94°C, 45 sec 50°C, 1 min 72°C; (3) 10 min 72°C final extension. The amplification of *MAT1-2* with degenerated primers used cycle conditions that differ only in two points: 35 sec at 72°C elongation time in each cycle and a total of 35 cycles. Finally, digested DNA prepared for inverse PCR was amplified under the following conditions: (1) 2 min 94°C; (2) 35 cycles of 40 sec 94°C, 45 sec 50°C, 90 sec 72°C; (3) 10 min 72°C for the final extension. In the end, all products obtained from degenerate primer PCR or inverse PCR were excised from the agarose gel and purified using the MinElute PCR Purification Kit (Qiagen) following the manufacturer’s recommendations.

### TAIL PCR for Sequencing the Flanking Regions

For sequencing the regions flanking the conserved regions of mating-type genes, three rounds of TAIL-PCR were performed with thermal cycling conditions as described in [44], with some modifications. Primary TAIL reaction (20 µl) contained 7.2 µl of Jump-Start, 0.2 µM of specific primer; 2 µM of respective degenerate primer and 20 ng of DNA. Degenerate primers used for HMG box motif were – TAIL-HMG-AD1, -AD5, -AD6 and for alpha box motif were TAIL-ALPHA-AD1 and -AD2 [45]. Cycling conditions were same as according to [44] with annealing reduced to 58°C and 55°C for high stringency cycles. For the secondary reactions, cycle number was increased to 15 with reduced annealing temperatures (58°C and 55°C) for high stringency cycles. The final round of TAIL-PCR was performed in 50 µl reaction volume. Products were purified and sequenced on one strand with the specific primer. After each round, PCR products were diluted (40× of primary TAIL-PCR was used as template for secondary and tenfold dilution of secondary was used for tertiary TAIL-PCR), and 1 µl of the diluted reaction mixture was used as template for next PCR.

### Sequencing

All PCR products were labeled with the Big Dye Terminator v3.1 Cycle Sequencing Kit (Life Technologies, Carlsbad, CA, USA) and cycle sequenced as follows: (1) 1 min 96°C; (2) 26 cycles of 20 sec 96°C, 5 sec 50°C and 2 min 60°C. Products were then purified using the Big Dye XTerminator Purification Kit (Life Technologies). Sequences were detected on 3130×l Genetic Analyzer (Life Technologies) and analyzed with the Sequencing Analysis Software 3.4 (Life Technologies).

### Multiplexing

Specific primers were designed using Primer 3 plus online tool [46] (Table 2). These primers were then checked for suitability for use in multiplex PCR. Primer pair Lpul_MAT1-2invFP1 and -RP1 along with primer set Lpul_MAT1-1F and R was used in multiplex PCR. The PCR mix used 20 ng DNA extract in a total volume of 50 µl containing 18 µl of JumpStart REDTaq ReadyMix PCR Reaction Mix (Sigma-Aldrich) and 200 nM of each primer. Cycling conditions were: (1) 2 min 94°C; (2) 35 cycles of 30 sec 94°C, 30 sec 54°C, 40 sec 72°C; (3) 10 min at 72°C final extension. *MAT1-1* specific primers amplified a product of ca. 380 bp, and *MAT1-2* primers amplified an 180 bp fragment. Axenic fungal culture DNA was used as positive control, whereas algal DNA and no template reactions served as negative controls.

### Population Genetic Analyses

Samples used in this study were collected in the framework of an earlier project (see Table 1) [35], [40], [47]. Eight fungal-specific and seven algal-specific microsatellite markers were amplified from total lichen DNA (for RTL, RVZ and RXS seven fungal specific and seven alga specific markers were used) [42], [47]–[49]. Fragment lengths were detected on a 3730xl DNA Analyzer (Life Technologies), and electropherograms were analyzed with GENEMAPPER 3.7 (Life Technologies) using LIZ500 as size standard. Multilocus genotypes (MLG) were defined separately for the fungal (based on eight loci) and for the algal (based on seven loci) symbionts.

To check for recombination or clonality in the fungal symbiont within populations, we performed two tests: i) the overall index of association (*I^S^_A_*) as implemented in LIAN 3.1 [50] (see Table 1); ii) linkage disequilibrium between pairs of loci within each population using Genepop [51], http://genepop.curtin.edu.au/; Table S1. The significance of allelic associations between pairs of loci was assessed by carrying out Fischer’s exact test as implemented in Genepop 4 software [51].

Expected heterozygosity (H) for each population was estimated for the fungal symbiont using Arlequin software [52] (Table 1). We used rarefaction to produce estimates of allelic richness that are comparable among populations with different sample sizes. Allelic richness was calculated for random subsamples equal to the smallest sample size across populations (10 samples) using ADZE [53].

For each population we calculated the number of unique multilocus genotypes (MLG) and their ratio in the population (G:N, N = number of genotyped specimens). The calculations were performed for fungal MLGs i.e. FG (FG:N) and algal MLGs i.e. AG (AG:N). Samples having identical MLGs were treated as clones. The degree of clonality of the fungus or percent clonality (CF) was derived from FG:N as follows: one hundred minus the percent of unique genotypes. As clonally derived lichen offspring are expected to share fungal and algal components genetically identical to their mother thallus (vertical photobiont transmission), we calculated the number of unique lichen MLG i.e. LG (LG:N, by combining fungal and algal MLG data) for each population.

We analyzed the ratio of MAT1-1, MAT1-2 isolates in all the populations (Table 1). As clonal amplification may skew mating-type frequencies, we accounted for this possibility by clone correcting populations according to haplotypes. We tested whether there was significant relationship between *MAT* gene imbalance and clonality, by performing a linear regression analysis [54] for *MAT* gene ratio (i.e. absolute difference between numbers of MAT1-1 and MAT1-2 individuals in a population divided by number of samples) versus percent clonality.

## Results

### Partial Characterization of the Mating-type Genes of *L. pulmonaria*

Degenerate primers (RsMat1-1F&R) [55] targeting the conserved alpha box motif of *MAT1-1* resulted in the amplification of approximately 500 bp fragment. Combining this sequence with the 454 sequence data we obtained a total of 900 bp sequence of *MAT1-1.* tBLASTx showed the presence of a conserved alpha box motif and high homology with MAT1-1 protein of various ascomycetes fungi: *Xanthoria polycarpa* (32%), *Fusarium pseudograminearum, Fusarium lunulosporum, Fusarium culmorum* (30%), *Aspergillus parasiticus*, *A. flavus*, *A. oryzae* (34%). The *L. pulmonaria*-specific *MAT1-1* α-box primers target a segment of approximately 380 bp. No band of this size was amplified in axenic photobiont DNA. Sequences obtained by degenerate PCR were then searched in the 454 data from the cultured *L. pulmonaria* mycobiont. Combining the degenerate PCR sequence with the 454 data and TAIL-PCR we obtained a 2666 bp fragment of the *MAT1-1* gene. No *MAT1-2* sequences were retrieved from the 454 data.

The conserved region of *MAT1-2*, i.e. the HMG box, was amplified from genomic DNA as well as from axenic mycobiont culture of *L. pulmonaria* with degenerate primers (ChHMG1 and ChHMG2). A band of the expected size (280–300 bp) was excised from the gel, purified and sequenced. Combining degenerate PCR and inverse PCR we obtained a total of 450 bp of the *MAT1-2* gene of *L. pulmonaria*. The sequenced region showed high similarity with lichen-forming ascomycetes and other fungi, e.g., *Xanthoria polycarpa, Xanthoria parietina* and *Xanthoria elegans (*83% similarity), *Xanthoria flammea* (79%), *Arthroderma simii* (75%), *Cladonia galindezii* (74%), *Aspergillus parasiticus* (70%). The translated protein BLAST showed the presence of a conserved HMG box. No band of this size was amplified in axenic photobiont DNA. The *L. pulmonaria MAT1-2* specific primers were then designed from this 450 bp region for multiplex PCR and TAIL-PCR. The newly-designed *L. pulmonaria*-specific *MAT1-2* primers amplified a fragment of 180 bp and were used in the multiplex PCR along with *L. pulmonaria* specific *MAT1-1* primer. We obtained *a total of 1400* *bp of MAT1-2 from L. pulmonaria by* combining the sequencing results of degenerate, inverse and TAIL PCR.

Sequences for the *MAT1-1* and *MAT1-2* mating-type genes in *L. pulmonaria* are deposited in GenBank Accession Nos. JX520967 (*MAT1-1*), JX520966 *(MAT1-2*).

### Mating-type Population Analysis and Genetic Signature of Recombination

In majority of the samples (96.03%) only one mating-type gene per specimen was amplified in the multiplex PCR of *L. pulmonaria*, i.e., single thalli without apothecia amplified either *MAT1-1* or *MAT1-2*, hence confirming *L. pulmonaria* to be heterothallic. The multiplex primers were used to determine the frequency of *MAT1-1* and *MAT1-2* in different populations of *L. pulmonaria* (Fig S1). We tested the null hypothesis of an equal number of MAT1-1 and MAT1-2 isolates within 27 *L. pulmonaria* populations from a broad geographic range for a total of 933 lichen thalli. Both mating types were found in all populations tested. However, in several populations we found significant deviations from the expected 1∶1 idiomorph ratio. Four populations were mostly formed by MAT1-1 individuals and nine populations of MAT1-2 individuals, out of the 27 investigated populations (Table 1). Deviations from expected ratio were observed in small as well as large populations and less clonal or highly clonal populations. When samples were clone-corrected based on fungal haplotypes, three populations were skewed towards *MAT1-2* (MO1 Norway, EG Spain, SA6 South Africa). Three populations were excluded from these analyses because 37 individuals (3.9% of total number of samples) amplified simultaneously with both *MAT1-1* and *MAT1-2* primers. These populations will be the subjects of further investigations (Singh et al., unpublished data).

We characterized population genetic diversity of 933 thalli of *L. pulmonaria* from 27 populations (Table 1), using eight fungal- and seven algal specific microsatellite markers. The genetic diversity of populations was assessed by calculating expected heterozygosity (H) and allelic richness as implemented in ADZE. We did not find significant differences in genetic diversity among populations, except for CHS, which displayed very low expected heterozygosity (H = 0.134) and allelic richness (1.598). The highest H was found in MO (0.584) while the highest allelic richness was found in EG (4.079). Average expected heterozygosity was 0.4233 and average allelic richness was 3.136.

The degree of clonality, assessed by calculating FG:N, differed significantly between populations, ranging from 10.14% (RXS) to 88.88% (CHS) with an overall average of 0.4450 (Table 1). The index of association (*I^S^_A_*) was significantly different from 0 in most of the populations (Table 1). Investigations of pairwise LD provide information about the intensity and extent of recombination in genomes. The proportion of the 28 possible pairs displaying significant pairwise LD ranged from 7.143% in RVZ to 82.142% in NT (Table S1). These findings were in concordance with the number of clonal lichen thalli accounting for pairs of thalli carrying genetically identical symbionts (Table 1). Linear regression analysis showed no relationship between *MAT* gene imbalance and clonality in populations (R-square = 0.00154, Fig. S2).

## Discussion

### Partial Characterization of *MAT* Loci in *L. pulmonaria*

Previous studies suggested that *L. pulmonaria* is a heterothallic species [38]. Our study provides genetic evidence of heterothallism in *L. pulmonaria*, i.e. the existence of a dimictic mating system with two idiomorphs, *MAT1-1* and *MAT1-2* located on a single locus. The majority of isolates (96.03%) of *L. pulmonaria* thalli amplified with either *MAT1-1* or *MAT1-2* specific primers.

The two idiomorphs were amplified using degenerate primers designed against conserved alpha box motif (*MAT1-1)* and HMG box motif (*MAT1-2*). The sequenced regions for both *MAT1-1* and *MAT1-2* showed presence of respective conserved motifs and also displayed high sequence similarity with mating-type genes of lichen-forming ascomycetes and other fungi.

### *MAT* Idiomorph Distribution

Recent population genetic studies have emphasized the crucial contribution of sexual reproduction in shaping the genetic composition of *L. pulmonaria* populations [56] and the obligate association of *L. pulmonaria* with the green algal photobiont *Dictyochloropsis reticulata* [35]. Previous studies also highlighted the limited dispersal capability of vegetative propagules [33], [57]–[58], thus suggesting sexual reproduction to be the dominant mode of gene flow among populations. The relative contribution of asexual versus sexual reproduction to the life cycle of *L. pulmonaria,* is not known. However the high number of different fungal-algal genotypic combinations suggests that the lichen is able to maintain the sexual pathway in the studied populations. In this lichen, fertility was found to be related to thallus size [59], a trend that has also been observed in other species of lichen-forming fungi [60]–[61]. This is in concordance with the ‘tangled bank’ theory [62] predicting that sexual reproduction is favored in biologically saturated environments (i.e., when density and competition increase) because it increases the number of niches by generating genetic diversity and allows the formation of new genotypic combinations of fungal and algal symbionts. As it has been shown for other symbiotic systems, e.g., coral hosts and their associated *Symbiodinium* strains, horizontal transmission maintains a higher diversity and thus ensures the survival of the symbiosis under different environmental conditions [63]. However, the acquisition of a new photobiont from environmental pools may fail, especially when host selectivity is high [64]. This is indeed the case in *L. pulmonaria*, where the fungus is highly selective towards its photobiont being associated with only one algal species that has not yet been found as free-living [65]. In *L. pulmonaria* apothecia usually occur at later life stages i.e. 15–25 years [41], [66], and at moist and well-lit sites [41]. Therefore, the species mainly depends on clonal reproduction (vertical transmission) especially at the early stages of population establishment, dispersing fungal and algal symbionts together. The presence of both mating types in all studied populations and the high number of different fungal-algal genotypic combinations suggest that the lichen is able to maintain the sexual pathway in the studied populations. However the significantly disproportionate representation of the two mating types in a population may hinder sexual reproduction, thus inhibiting the species from taking the advantage of recombination even after maintaining the sexual cycle.

Our analyses of 27 populations of *L. pulmonaria* showed that strains of both mating types are present in all populations. However, we found skewed MAT isolate ratios in 13 out of 27 populations tested. We used several population genetics tools to test for the occurrence of recombination, for further inferring the mode of reproduction of the studied populations. The expected heterozygosity in the populations ranged from 0.134 (pop: CHS) to 0.584 (pop: MO1) with an average of 0.4233. The percentage of clonality ranged from 10.14% in RXS to 88.88% in CHS. This indicates that all of the studied populations utilize sexual as well as asexual reproductive strategies for their propagation. However the prevalence varies in different populations, with some populations mostly asexual and others more recombinant. For example, the Swiss population CHS, German population DS1 and Greek population GRR have high index of association with 88.88%, 70.42% and 77.14% clonality respectively. Mating gene analysis in these populations showed skewed *MAT* genes ratio. Since *L. pulmonaria* is a heterothallic species and our analyses showed significantly unbalanced *MAT* gene distribution in these populations, the observed high clonality in these populations may be caused by the lack or paucity of compatible spermatia (see Fig 7 in [38]). Conservation strategies for these populations should aim to equilibrate mating-type frequency and spatial distribution during conservation measures, to maximize the chances of sexual reproduction.

Certain populations, e.g. RTL (Russia), SA6 (South Africa), EG (Spain) and CA (Ukraine) displayed low clonality but medium to high genetic diversity (H = 0.538–0.402), low LD and low index of association (Ia), suggesting past sexual history. One population MO1 (Norway), showed average LD and Ia, in-spite of low clonality and high genetic diversity. However current mating-type gene ratio in these populations was profoundly unbalanced. This may be the result of stochastic events such as genetic drift or population bottleneck resulting in deficit of a particular mating type in a population in recent past, thus still having high genetic diversity. The present scenario of biased representation of either mating type in such populations may hinder sexual reproduction in this heterothallic species. Special attention for conservation should be given to such populations. Thallus translocations aiming to restore the mating-type gene balance should be tested in future as a conservation measure.

Population EGR showed the lowest genetic diversity (H) and low Ia, with 44.44% clonality. This population is small (nine individuals) and has biased *MAT* gene ratio. It is sensitive to decline or even extinction because of the scarcity of compatible MAT type isolates (only one MAT1-1 individual), combined with small population size and low genetic diversity. The appropriate conservation strategy for such populations would be thallus translocations aiming not only to increase the population size but also to balance the *MAT* gene ratio.

Our results show that clonality in populations cannot be used to predict *MAT* gene imbalance in natural populations. This is probably due to a number of factors that i) alter the genetic makeup of populations over time independently of changes in their *MAT* gene composition (e.g. gene flow, mutation, genetic drift) [67]), or ii) introduce bias in the analysis of the clonal structure of populations (random sampling of the finite population and suboptimal resolving power of the chosen genetic markers) [68]). In addition *L. pulmonaria* sexual reproduction usually occurs at later life stages (15–25 years) [41], [66] before which it remains clonal. During these long years of population establishment and propagation clonality is the prime mode of reproduction. Depending upon the population history and founder individuals *MAT* genes can be balanced or dis-balanced independent of the observed clonality of the population. Significance of *MAT* gene frequency is apparent only after many years of population establishment and long clonal history, which might have resulted in current disproportionate representation of mating-type isolates in the population. Availability of reliable markers for mating type identification in such species thus can be used to estimate current MAT isolate distribution in such populations. Furthermore thallus transplants of scarce mating-type isolate in a population will increase the probability of successful sexual reproduction and hence long distance spore dispersal. The main conclusion we can draw from these results is that, at least in case of natural populations of lichen-forming fungi such as *Lobaria pulmonaria* with long generation time and clonal history along with delayed sexual reproduction, clonality cannot be used to predict *MAT* imbalance.

### Putative Conversion to Homothallism

A few samples (3.9% of the total) from three populations showed amplification with both mating-type markers. We exclude primer or sample contamination, as PCRs were double-checked in the presence of positive - (mycobiont culture DNA), negative - (photobiont DNA) and no-template controls. Since all apothecia were removed prior to DNA extraction, we can exclude the amplification of DNA from intra-thalline spores as an explanation. Therefore we envision three possible explanations to account for this observation: i) presence of conidia (asexual fungal spores), or vegetative diaspores (asexual lichen propagules) of an individual with opposite mating type on the lobe that was used for extraction; ii) fusion of thalli with two opposite mating types during the formation of a new lichen thallus (redifferentiation; [4], [69]) iii) co-occurrence of *MAT1-1* and *MAT1-2* in the same thallus, i.e. local switch to homothallism. Concerning the first two points, we can exclude the amplification of a DNA mix from different thalli or vegetative diaspores as this would have resulted in double peaks at some or all *L. pulmonaria*-specific microsatellite loci. None of the samples from the three populations having both *MAT1-1* and *MAT1-2* showed the presence of double peaks at any of the eight fungal-specific microsatellite loci.

Intriguingly, there are reports of inter-conversion and co-occurrence of both kind of sexual systems, i.e. homothallism and heterothallism, within the same species. Some filamentous ascomycetes have been reported to be conditionally homothallic under specific environmental conditions, e.g. the ascomycete *Candida albicans* [70], the basidiomycete *Cryptococcus neoformans* [71]–[72]. The transition from heterothallism may lead to reshuffling of genes even in unigenotype populations.

In our study, the co-occurrence of both idiomorphs in the same lichen thallus was restricted to a few populations that require further studies. The switch to homothallism could offer a choice between outcrossing and selfing in a population, the relative frequency of their occurrence being governed by specific environmental and genetic conditions [73]–[74]. Thus, depending upon the external triggers one or the other breeding strategy may be favored. Although we can exclude the presence of mix DNA the switch to homothallism needs to be confirmed in two ways: i) sequencing the mating-type locus of a single-spore mycobiont culture should retrieve both idiomorphs; ii) studies of gene expression should show the co-expression of *MAT1-1* and *MAT1-2* in the same lichen thallus.

### Conclusions and Implications for Conservation

Earlier studies suggested conservation translocations through thallus transplants for *L. pulmonaria* as a measure for conservation, especially for small and isolated populations [75]–[76]. These studies gave conservation preference to sexual populations rather than asexual ones on the account of higher genetic variation in sexual populations and importance of sexual reproduction in adaptation and evolution. The higher dispersal range of sexual spores as compared to vegetative spores may also lead to long distance dispersal and acquisition of new habitat.

This study provides important insights for improving the conservation strategies of this threatened lichen species and provides guidelines for other heterothallic lichen-forming fungi. Previous studies provide evidence that the balance between sexual and asexual reproduction in fungi may shift in response to selective pressures [77]–[78]. Considering the importance of sexual reproduction for adaptation and evolution, we suggest that conservation translocations of thallus transplants shall also consider the frequency and local distribution of MAT isolates, in addition to increasing the number of genotypes. This strategy would aim at: i) maintaining recombinant those locally adapted gene pools recently identified by co-phylogeographic studies on *L. pulmonaria* symbionts [38], [40], [79] and, ii) balancing the *MAT* idiomorph ratio towards equal frequencies of the mating types. The mating-type predetermined thallus transplant can also be used to balance the ratio in populations with skewed idiomorph ratio. This will increase the probability of sexual reproduction due to increased availability of spermatia of the compatible mating type. Skewed frequencies and incompletely overlapping spatial distribution of mating-type idiomorphs in a population can limit sexual reproduction and thus be limiting for adaptation and also for exploration of new habitats for re-colonization. Implementing conservation translocation strategies in due consideration of *MAT* genotypes may help prevent small and fragmented populations from extinction by providing an option of “switching to sexual mode” when facing changing environmental conditions. This mating type-specific conservation translocation can hence assist in increasing genetic diversity based on the available allelic variation in the population, thus enhancing adaptation. Furthermore, previous studies have shown that even rare events of sexual reproduction may suffice to alter lichen population genetic structure [35], [80] as horizontal photobiont transmission can reshuffle the symbiosis and generate better adapted fungal-algal pairs [35].

## Supporting Information

Figure S1 Gel image showing multiplex-PCR of eight random samples of *L. pulmonaria* using *L. pulmonaria*-specific *MAT1-1* and *MAT1-2* primers.
(TIFF)

Figure S2 Scatter plot displaying the relationship between *MAT gene* ratio (i.e. absolute difference between numbers of MAT1-1 and MAT1-2 individuals in a population divided by number of samples) and percent clonality in 27 natural populations of *L. pulmonaria.*
(PDF)

Table S1 Linkage disequilibrium map for the eight fungal microsatellite loci in 29 populations used in this study. Significantly linked loci pairs (p<0.05) are indicated by shaded boxes.
(XLSX)

## References

[1] Bell G (1982) The Masterpiece of Nature: The Evolution and Genetics of Sexuality. Berkeley: University of California Press. 635 p.

[2] Whittle CA, Nygren K, Johannesson H (2011) Consequences of reproductive mode on genome evolution in fungi. Fungal Genet Biol 48: 661–667. 21362492 10.1016/j.fgb.2011.02.005

[3] Scarascia-Mugnozza GT, Perrino P (2002) The history of ex-situ conservation and use of plant genetic resources. In: Engels JMM, Ramanatha Rao V, Brown AHD, Jackson MT, editors. Managing plant genetic diversity. Oxon: CABI Publishing. 1–22.

[4] Murtagh GJ, Dyer PS, Crittenden PD (2000) Sex and the single lichen. Nature 404: 564. 10766229 10.1038/35007142

[5] Honegger R, Scherrer S (2008) Sexual reproduction in lichen-forming ascomycetes. In: Nash TH III, editor. Lichen Biology. Cambridge: Cambridge University Press. 94–103.

[6] Büdel B, Scheidegger C (2008) Thallus morphology and anatomy. In: Nash TH III, editor. Lichen Biology. Cambridge: Cambridge University Press. 40–68.

[7] Seymour FA, Crittenden PD, Dickinson MJ, Paoletti M, Montiel D, et al (2005) Breeding systems in the lichen-forming fungal genus *Cladonia* . Fungal Genet Biol 42: 554–63. 15893256 10.1016/j.fgb.2005.03.006

[8] Charlesworth B (1980) The cost of sex in relation to mating system. J Theor Biol 84: 655–671. 7431946 10.1016/s0022-5193(80)80026-9

[9] Charlesworth B (1990) Mutation-selection balance and the evolutionary advantage of sex and recombination. Genet Res 55: 199–221. 2394378 10.1017/s0016672300025532

[10] Kück U, Pöggeler S (2009) Cryptic sex in fungi. Fungal Biol Rev 23: 86–90.

[11] Hawksworth DL (1990) The long-term effects of air pollutants on lichen communities in Europe and North America. In: Woodwell GM, editor. The Earth in transition: patterns and processes of biotic impoverishment. Cambridge: Cambridge University Press. 45–64.

[12] Brown DH (1992) Impact of agriculture on bryophytes and lichens. In: Bates JW, Farmer AM, editors. Bryophytes and lichens in a changing environment. Oxford: Clarendon Press. 259–83.

[13] Hawksworth DL, Rose F, Coppins BJ (1973) Changes in the lichen flora of England and Wales attributable to pollution of the air by sulphur dioxide. In: Ferry BW, Baddeley MS, Hawksworth DL, editors. Air pollution and lichens. Toronto: University Toronto Press. 330–367.

[14] Stofer S, Bergamini A, Aragon G, Carvalho P, Coppins BJ, et al (2006) Species richness of lichen functional groups in relation to land use intensity. Lichenologist 38: 331–353.

[15] Coppin E, Debuchy R, Arnaise S, Picard M (1997) Mating types and sexual development in filamentous ascomycetes. Microbiol Mol Biol Rev 61: 411–428. 9409146 10.1128/mmbr.61.4.411-428.1997 PMC232618

[16] Kronstad JW, Staben C (1997) Mating type in filamentous fungi. Annu Rev Genet 31: 245–276. 9442896 10.1146/annurev.genet.31.1.245

[17] Heitman J, Kronstad JW, Taylor JW, Casselton LA (2007) Sex in Fungi: Molecular Determination and Evolutionary Implications. Washington DC: ASM Press. 572 p.

[18] Turgeon BG, Yoder OC (2000) Proposed nomenclature for mating type genes of filamentous ascomycetes. Fungal Genet Biol 31: 1–5. 11118130 10.1006/fgbi.2000.1227

[19] Nelson MA (1996) Mating systems in Ascomycetes: a romp in the sac. Trends Genet 12: 69–74. 8851974 10.1016/0168-9525(96)81403-x

[20] Turgeon BG, Bohlmann H, Ciuffetti LM, Christiansen SK, Yang G, et al (1993) Cloning and analysis of the mating type genes from *Cochliobolus heterostrophus* . Mol Gen Genet 238: 270–284. 8479433 10.1007/BF00279556

[21] Martin T, Lu S-W, van Tilbeurgh H, Ripoll DR, Dixelius C, et al (2010) Tracing the origin of fungal α1 domain places its ancestor in the HMG-box superfamily: Implications for fungal mating-type evolution. PLoS ONE 5: e15199 doi:10.1371/journal.pone.0015199. 21170349 10.1371/journal.pone.0015199 PMC2999568

[22] Arie T, Christiansen SK, Yoder OC, Turgeon BG (1997) Efficient cloning of ascomycete mating type genes by PCR amplification of the conserved *MAT* HMG box. Fungal Genet Biol 21: 118–130. 9126621

[23] Arie T, Kaneko I, Yoshida T, Noguchi M, Nomura Y, et al (2000) Mating-type genes from asexual phytopathogenic ascomycetes *Fusarium oxysporum* and *Alternaria alternata* . Mol Pl Micr Interact 13: 130–1339. 10.1094/MPMI.2000.13.12.1330 11106025

[24] Honegger R, Zippler U, Gansner H, Scherrer S (2004) Mating systems in the genus *Xanthoria* (lichen-forming ascomycetes). Mycol Res 108: 480–488. 15230000 10.1017/s0953756204009682

[25] Rydholm C, Dyer PS, Lutzoni F (2007) DNA sequence characterization and molecular evolution of *MAT1* and *MAT2* mating-type loci of the self-compatible ascomycete mold *Neosartorya fischeri* . Eukaryot Cell 6: 868–874. 17384199 10.1128/EC.00319-06 PMC1899244

[26] Dyer PS, Paoletti M (2005) Reproduction in *Aspergillus fumigatus*: sexuality in a supposedly asexual species? Med Mycol 43: S7–S14. 16110786 10.1080/13693780400029015

[27] Consolo V, Cordo C, Salerno G (2005) Mating-type distribution and fertility status in *Magnaporthe grisea* populations from Argentina. Mycopathologica 160: 285–290. 10.1007/s11046-005-4333-3 16244896

[28] Saleh D, Xu P, Shen Y, Li C, Adreit H, et al (2012) Sex at the origin: an Asian population of the rice blast fungus *Magnaporthe oryzae* reproduces sexually. Mol Ecol 21: 1330–44. 22313491 10.1111/j.1365-294X.2012.05469.x

[29] Olarte RA, Horn BW, Dorner JW, Monacell JT, Singh R, et al (2012) Effect of sexual recombination on population diversity in aflatoxin production by *Aspergillus flavus* and evidence for cryptic heterokaryosis. Mol Ecol 21: 1453–76. 22212063 10.1111/j.1365-294X.2011.05398.x

[30] Grube M, Spribille T (2012) Exploring symbiont management in lichens. Mol Ecol 21: 3098–3099. 22916345 10.1111/j.1365-294x.2012.05647.x

[31] Hilmo O, Lundemo S, Holien H, Stengrundet K, Stenoien HK (2012) Genetic structure in a fragmented Northern Hemisphere rainforest: large effective sizes and high connectivity among populations of the epiphytic lichen *Lobaria pulmonaria.* . Mol Ecol 21: 3250–3265. 22571538 10.1111/j.1365-294X.2012.05605.x

[32] Schei FH, Blom HH, Gjerde I, Grytnes JG, Heegaard E (2012) Fine-scale distribution and abundance of epiphytic lichens: environmental filtering or local dispersal dynamics? J Veg Sci 23: 459–470.

[33] Werth S, Wagner HH, Gugerli F, Holderegger R, Csencsics D, et al (2006) Quantifying dispersal and establishment limitation in a population of an epiphytic lichen. Ecology 87: 2037–2046. 16937643 10.1890/0012-9658(2006)87[2037:qdaeli]2.0.co;2

[34] Scheidegger C, Werth S (2009) Conservation strategies for lichens: insights from population biology. Fung Biol Rev 23: 55–66.

[35] Dal Grande F, Widmer I, Wagner HH, Scheidegger C (2012) Vertical and horizontal photobiont transmission within populations of a lichen symbiosis. Mol Ecol 21: 3159–72. 22384938 10.1111/j.1365-294X.2012.05482.x

[36] Wirth VHS, Schöller H, Scholz P, Ernst G, Feuerer T, et al (1996) Rote Liste der Flechten (Lichenes) der Bundesrepublik Deutschland. In: Bonn-Bad Godesberg: Bundesamt für Naturschutz, Schriftenreihe für Vegetationskunde Schmitt JA, Benkert D, Dörfelt H, editors. Rote Liste Gefährdeter Pflanzen Deutschlands. 28: 307–368.

[37] Scheidegger C, Goward T (2002) Monitoring lichens for conservation: red lists and conservation action plans. In: Nimis PL, Scheidegger C, Wolseley P, editors. Lichen Monitoring - Monitoring Lichens. Dordrecht: Kluwer Academic Publishers. 163–181.

[38] Zoller S, Lutzoni F, Scheidegger C (1999) Genetic variation within and among populations of the threatened lichen *Lobaria pulmonaria* in Switzerland and implications for its conservation. Mol Ecol 8: 2049–2059. 10632856 10.1046/j.1365-294x.1999.00820.x

[39] Selkoe KA, Toonen RJ (2006) Microsatellites for ecologists: a practical guide to using and evaluating microsatellite markers. Ecology Letters 9: 615–629. 16643306 10.1111/j.1461-0248.2006.00889.x

[40] Widmer I, Dal Grande F, Excoffier L, Holderegger R, Keller C, et al. (2012) European phylogeography of the epiphytic lichen fungus *Lobaria pulmonaria* and its green algal symbiont. Mol Ecol DOI: 10.1111/mec.12051. 10.1111/mec.12051 23094600

[41] Denison WC (2003) Apothecia and ascospores of *Lobaria oregana* and *Lobaria pulmonaria* investigated. Mycologia 95: 513–518. 21156641

[42] Widmer I, Dal Grande F, Cornejo C, Scheidegger C (2010) Highly variable microsatellite markers for the fungal and algal symbionts of the lichen *Lobaria pulmonaria* and challenges in developing biont-specific molecular markers for fungal associations. Fungal Biol 114: 538–544. 20943165 10.1016/j.funbio.2010.04.003

[43] Ochman H, Gerber AS, Hartl DL (1988) Genetic applications of an inverse polymerase chain reaction. Genetics 120: 621–623. 2852134 10.1093/genetics/120.3.621 PMC1203539

[44] Liu YG, Whittier RF (1995) Thermal Asymmetric Interlaced PCR - automatable amplification and sequencing of insert end fragments from P1 and YAC clones for chromosome walking. Genomics 25: 674–681. 7759102 10.1016/0888-7543(95)80010-j

[45] Singer T, Burke E (2003) High-throughput TAIL-PCR as a tool to identify DNA flanking insertions. Methods Mol Biol 236: 241–271. 14501069 10.1385/1-59259-413-1:241

[46] Untergasser A, Nijveen H, Rao X, Bisseling T, Geurts R, et al (2007) Primer3Plus, an enhanced web interface to Primer3. Nucleic Acids Res 35: W71–74. 17485472 10.1093/nar/gkm306 PMC1933133

[47] Mikryukov VS (2011) Population ecology of epiphytic lichen *Lobaria pulmonaria* (L.) Hoffm. in Urals and Siberia. Ph.D. Thesis. Institute of Plant and Animal Ecology, Ural Branch, Russian Academy of Sciences: Ekaterinburg, Russia.

[48] Walser JC, Sperisen C, Soliva M, Scheidegger C (2003) Fungus-specific microsatellite primers of lichens: application for the assessment of genetic variation on different spatial scales in *Lobaria pulmonaria* . Fungal Genet Biol 40: 72–82. 12948515 10.1016/s1087-1845(03)00080-x

[49] Dal Grande F, Widmer I, Beck A, Scheidegger C (2010) Microsatellite markers for *Dictyochloropsis reticulata* (Trebouxiophyceae), the symbiotic alga of the lichen *Lobaria pulmonaria* (L.). Conserv Genet 11: 1147–1149.

[50] Haubold B, Hudson RR (2000) LIAN 3.0: detecting linkage disequilibrium in multilocus data. Bioinformatics 16: 847–848. 11108709 10.1093/bioinformatics/16.9.847

[51] Raymond M, Rousset F (1995) genepop (Version 1.2): population genetics software for exact tests and ecumenicism. J Hered 86: 248–249.

[52] Excoffier L, Laval G, Schneider S (2005) Arlequin ver. 3.0: An integrated software package for population genetics data analysis. Evol Bioinform Online 1: 47–50. PMC2658868 19325852

[53] Szpiech ZA, Jakobsson M, Rosenberg NA (2008) ADZE: a rarefaction approach for counting alleles private to combinations of populations. Bioinformatics 24: 2498–2504. 18779233 10.1093/bioinformatics/btn478 PMC2732282

[54] AnalystSoft Inc. StatPlus:mac - statistical analysis program for Mac OS. Version 2009. www.analystsoft.com/en/(accessed 17 October 2012).

[55] Foster SJ, Fitt BDL (2004) Isolation and characterization of the mating-type (*MAT)* locus from *Rhynchosporium secalis* . Curr Genet 44: 277–286. 10.1007/s00294-003-0445-9 14517690

[56] Walser JC, Gugerli F, Holderegger R, Kuonen D, Scheidegger C (2004) Recombination and clonal propagation in different populations of the lichen *Lobaria pulmonaria* . Heredity 93: 322–329. 15241450 10.1038/sj.hdy.6800505

[57] Walser JC (2004) Molecular evidence for limited dispersal of vegetative propagules in the epiphytic lichen *Lobaria pulmonaria* . Am J Bot 91: 1273–1276. 21653485 10.3732/ajb.91.8.1273

[58] Werth S, Gugerli F, Holderegger R, Wagner HH, Csencsics D, et al (2007) Landscape-level gene flow in *Lobaria pulmonaria*, an epiphytic lichen. Mol Ecol 16: 2807–2815. 17594449 10.1111/j.1365-294X.2007.03344.x

[59] Pentecost A, Richardson CB (2011) Niche separation and overlap in the foliose lichens *Lobaria pulmonaria* (L.) Hoffm. and *L. virens* (With.) Laundon in the Killarney oakwoods, Ireland. Royal Irish Academy 111: 1–7.

[60] Gauslaa Y, Palmqvist K, Solhaug KA, Hilmo O, Holien H, et al (2009) Size-dependent growth of two old-growth associated macrolichen species. New Phytol 181: 683–692. 19032441 10.1111/j.1469-8137.2008.02690.x

[61] Pringle A, Chen D, Taylor JW (2003) Sexual fecundity is correlated to size in the lichenized *Xanthoparmelia cumberlandia* . Bryologist 106: 221–225.

[62] Koella JC (1988) The Tangled Bank: The maintenance of sexual reproduction through competitive interactions. J Evol Biol 2: 95–116.

[63] Stat M, Loh WKW, Hoegh-Guldberg O, Carter DA (2008) Symbiont acquisition strategy drives host-symbiont associations in the southern Great Barrier Reef. Coral Reefs 27: 763–772.

[64] Genkai-Kato M, Yamamura N (1999) Evolution of mutualistic symbiosis without vertical transmission. Theor Popul Biol 55: 309–323. 10366555 10.1006/tpbi.1998.1407

[65] Dal Grande F (2011) Phylogeny and co-phylogeography of a photobiont-mediated guild in the lichen family Lobariaceae. PhD Thesis. Bern, Switzerland: University of Bern.

[66] Hoistad F, Gjerde I (2011) *Lobaria pulmonaria* can produce mature ascospores at an age of less than 15 years. Lichenologist 43: 495–497.

[67] Tomiuk J, Guldbrandtsen B, Loeschcke V (1998) Population differentiation through mutation and drift – a comparison of genetic identity markers. Genetica 102/103: 545–558. 9720297

[68] Arnaud-Haond S, Duarte CM, Alberto F, Serrão EA (2007) Standardizing methods to address clonality in population studies. Mol Ecol 16: 5115–5139. 17944846 10.1111/j.1365-294X.2007.03535.x

[69] Honegger R (2008) Mycobionts. In: Nash TH III, editor. Lichen Biology, 2nd edn. Cambridge: Cambridge University Press. 27–39.

[70] Alby K, Schaefer D, Bennett RJ (2009) Homothallic and heterothallic mating in the opportunistic pathogen *Candida albicans* . Nature 460: 890–893. 19675652 10.1038/nature08252 PMC2866515

[71] Lin X, Hull CM, Heitman J (2005) Sexual reproduction between partners of the same mating type in *Cryptococcus neoformans* . Nature 434: 1017–1021. 15846346 10.1038/nature03448

[72] Wang L, Lin X (2011) Mechanisms of unisexual mating in *Cryptococcus neoformans* . Fungal Genet Biol 48: 651–660. 21320625 10.1016/j.fgb.2011.02.001

[73] Scherrer S, Zippler U, Honegger R (2005) Characterization of the mating-type locus in the genus *Xanthoria* (lichen-forming ascomycetes, Lecanoromycetes). Fungal Genet Biol 42: 976–988. 16266815 10.1016/j.fgb.2005.09.002

[74] Paoletti M, Seymour FA, Alcocer MJC, Kaur N, Calvo AM, et al (2007) Mating type and the genetic basis of self-fertility in the model fungus *Aspergillus nidulans.* . Curr Biol 17: 1384–1389. 17669651 10.1016/j.cub.2007.07.012

[75] Scheidegger C (1995) Early development of transplanted isidioid soredia of *Lobaria pulmonaria* in an endangered population. Lichenologist 27: 361–374.

[76] Scheidegger C, Frey B, Zoller S (1995) Transplantation of symbiotic propagules and thallus fragments: methods for the conservation of threatened epiphytic lichen populations. Mitteilungen der Eidgenössisches Forschungsanstalt für Wald, Schnee und Landschaft 70: 41–62.

[77] Barton NH, Charlesworth B (1998) Why sex and recombination? Science 281: 1986–1990. 9748151

[78] Otto SP, Lenormand T (2002) Resolving the paradox of sex and recombination. Nat Rev Genet 3: 252–261. 11967550 10.1038/nrg761

[79] Scheidegger C, Bilovitz PO, Werth S, Widmer I, Mayrhofer H (2012) Hitchhiking with forests: population genetics of the epiphytic lichen *Lobaria pulmonaria* in primeval and managed forests in southeastern Europe. Ecology and Evolution 2: 2223–2240. 23139881 10.1002/ece3.341 PMC3488673

[80] Honegger R, Zippler U (2007) Mating systems in representatives of Parmeliaceae, Ramalinaceae and Physciaceae (Lecanoromycetes, lichen-forming ascomycetes). Mycol Res 111: 424–32. 17512182 10.1016/j.mycres.2007.02.005

