# Supplementary figures and images for: Genetic Basis of Self-Incompatibility in the Lichen-Forming Fungus Lobaria pulmonaria and Skewed Frequency Distribution of Mating-Type Idiomorphs: Implications for Conservation
Source: PLoS One. 2012 Dec 7;7(12):e51402. doi: 10.1371/journal.pone.0051402 (PMC3517546; doi:10.1371/journal.pone.0051402)

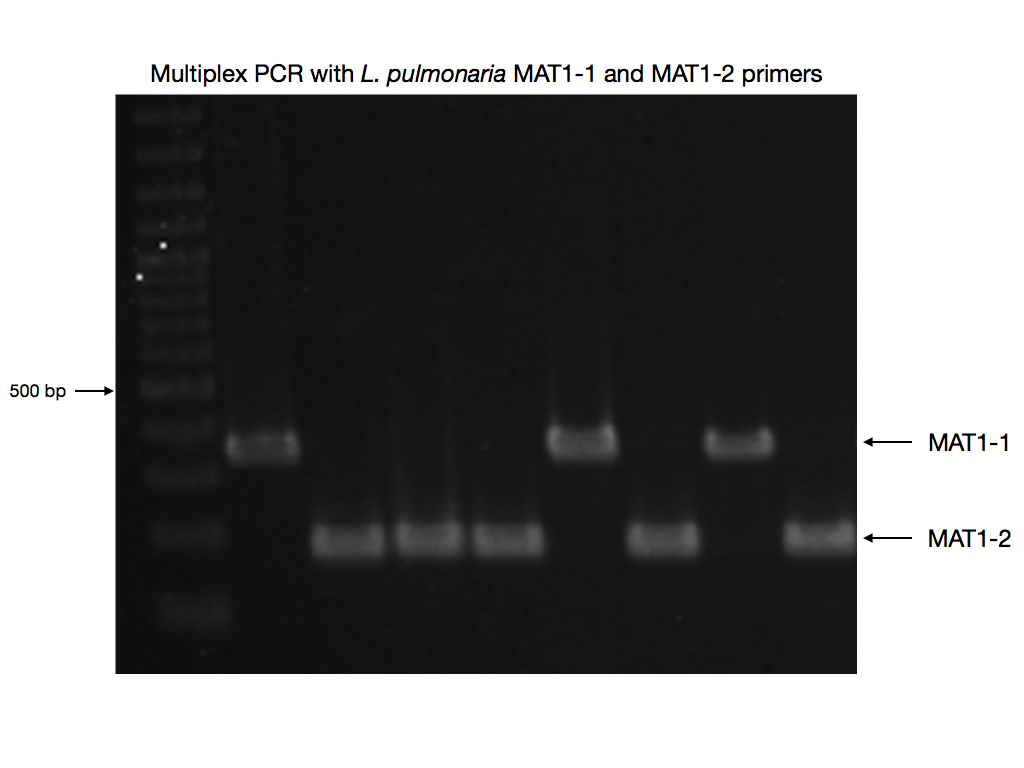

Supplement: Figure S1 — Gel image showing multiplex-PCR of eight random samples of L. pulmonaria using L. pulmonaria-specific MAT1-1 and MAT1-2 primers. (TIFF) [file pone.0051402.s001.tiff]

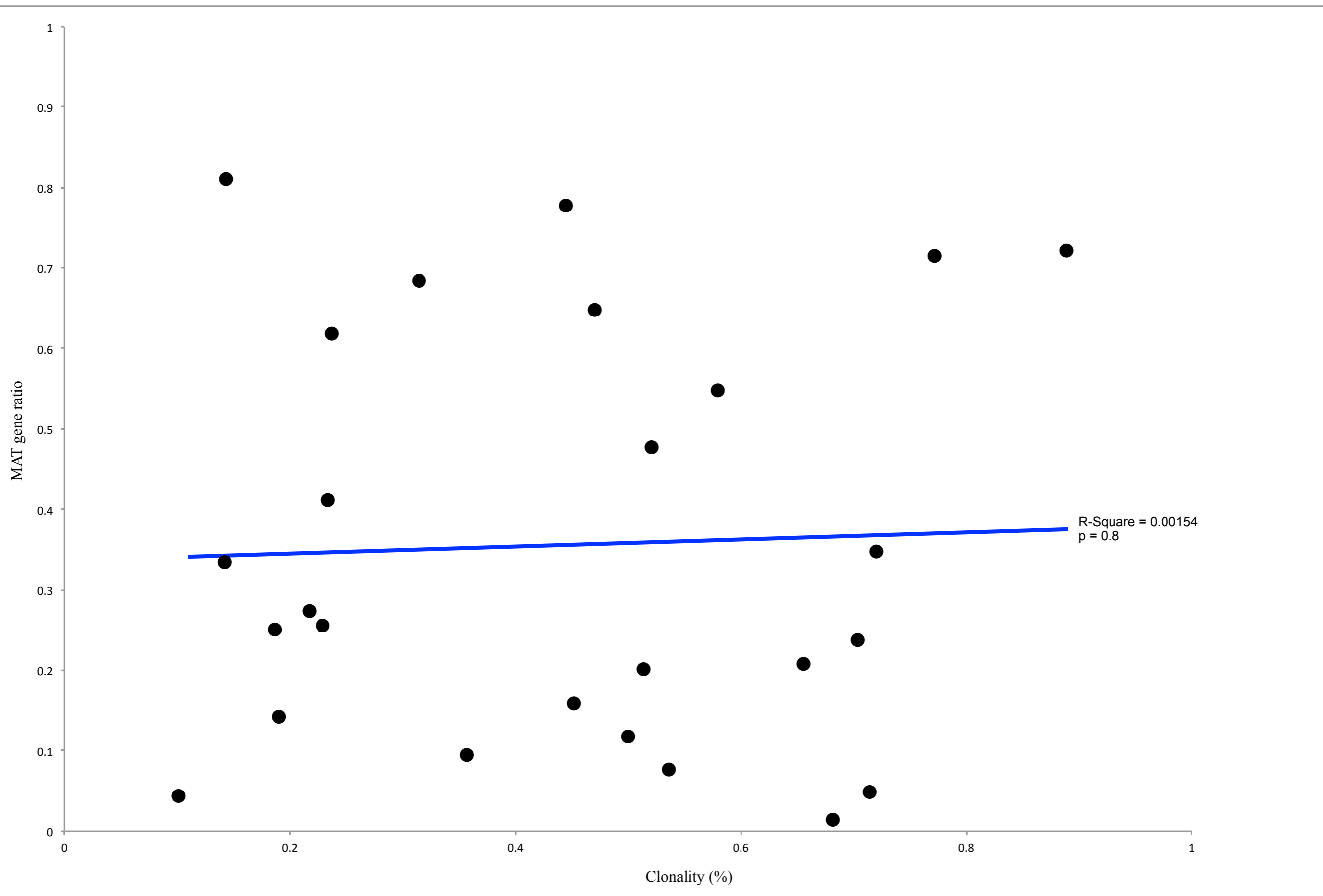

Supplement: Figure S2 — Scatter plot displaying the relationship between MAT gene ratio (i.e. absolute difference between numbers of MAT1-1 and MAT1-2 individuals in a population divided by number of samples) and percent clonality in 27 natural populations of L. pulmonaria. (PDF) [file pone.0051402.s002.pdf]
